# Fluidodinâmica Computacional (CFD) para Prever Alterações Patológicas na Aorta: Está Pronta para Uso Clínico?

**DOI:** 10.36660/abc.20220040

**Published:** 2022-02-14

**Authors:** Dominik Obrist, Hendrik von Tengg-Kobligk

**Affiliations:** 1 ARTORG Center for Biomedical Engineering Research University of Bern Bern Suíça ARTORG Center for Biomedical Engineering Research, University of Bern, Bern – Suíça; 2 Institute for Diagnostic, Interventional and Pediatric Radiology University of Bern Bern Suíça Institute for Diagnostic, Interventional and Pediatric Radiology (DIPR), University of Bern, Bern – Suíça

**Keywords:** Doenças Cardiovasculares/fisiopatologia, Doenças Cardiovasculares/diagnóstico, Simulação por Computador, Processamento de Imagem Assistida por Computador, Desenho de Próteses, Implantação de Prótese, Engenharia Biomédica

A modelagem computacional de problemas de fluxo complexos se beneficia de uma base já estabelecida de software de Fluidodinâmica Computacional (CFD, do inglês *Computational Fluid Dynamics* ) desenvolvido nas últimas décadas. O uso inicial de CFD estava limitado à pesquisa acadêmica. Atualmente, é uma ferramenta totalmente estabelecida em muitas aplicações industriais (por exemplo, automotiva, aeroespacial), embora a CFD continue sendo uma das tarefas computacionais mais exigentes e o estudo de problemas de fluxo complexos seja frequentemente limitado pelo poder computacional disponível. O aumento exponencial contínuo na capacidade computacional e a melhora da acessibilidade à infraestrutura de computação de alto desempenho foi um facilitador para o uso cada vez maior de CFD. À luz desse sucesso, é surpreendente que a CFD dificilmente seja encontrada na prática clínica. Apesar do notável progresso na modelagem do fluxo sanguíneo complexo e na identificação de marcadores quantitativos para padrões de fluxo patológico,^1^ a maior parte das aplicações biomédicas da CFD permanece ao nível de pesquisa acadêmica e casos de paciente único. O estudo de Almeida et al.,^2^ é um dos poucos estudos sobre CFD que utiliza dados radiológicos longitudinais de coortes de pacientes. A proposta de previsão de alterações patológicas na aorta com base na CFD ilustra o potencial dessa tecnologia para se tornar uma modalidade diagnóstica estabelecida. Alguns dos desafios abordados com sucesso neste estudo são exemplos que ilustram os motivos pelos quais a CFD ainda não encontrou seu lugar na rotina clínica. Pode-se identificar quatro campos de problemas: a) A dificuldade de gerar modelos eficientes de CFD específicos para o paciente; b) A necessidade de redução da complexidade dos dados para tornar os resultados da CFD acessíveis ao médico; c) A infraestrutura inexistente de TI que integra de maneira eficiente as ferramentas de CFD aos fluxos de trabalho de dados clínicos existentes; d) A falta de especialistas no ambiente clínico, ou seja, um engenheiro clínico que dê suporte aos médicos que cuidam dos pacientes. A seguir, discutiremos esses problemas e indicaremos possíveis medidas para mitigá-los.

## Modelagem específica para o paciente

Modelos específicos para pacientes^3^ foram implementando com sucesso por muitos pesquisadores. No entanto, a tradução de dados radiológicos de alta resolução em modelos vasculares geométricos para CFD continua sendo uma tarefa demorada, muitas vezes trabalhosa e requer especialistas treinados. Há uma falta de ferramentas automatizadas de segmentação e mesclagem para tarefas biomédicas. Desenvolvimentos recentes baseados em redes neurais profundas podem fornecer soluções aceitáveis para uso clínico.^4^

A definição das condições de contorno (por exemplo, perfis de velocidade no *inflow* / *outflow* ) requer muito cuidado, pois as condições de contorno têm um forte efeito na qualidade dos resultados. Portanto, é útil incluir medidas de fluxo em locais bem definidos (por exemplo, RM de fluxo sanguíneo 2D+time ou 3D+time) em protocolos radiológicos.^5^ Além da escolha adequada da modalidade e localização no corpo, isso requer resolução temporal e espacial adequada dos exames.

Além disso, precisamos de uma melhor compreensão da biomecânica do tecido doente para interpretar corretamente uma aorta remodelada ou dissecada ou a placa na parede de um vaso. Além da pesquisa biomecânica clássica contínua, isso requer estudos com grandes coortes, incluindo voluntários saudáveis, para entender a relevância clínica dos modelos biomecânicos.

## Marcadores diagnósticos

A análise dos resultados de CFD é um desafio, mesmo para especialistas em dinâmica de fluidos. Isso se deve em parte à dificuldade em visualizar campos de fluxo tridimensionais dependentes do tempo que apresentam uma riqueza de fenômenos de fluxo que podem ser relevantes para a interpretação clínica dos resultados (por exemplo, vórtices, instabilidades de fluxo, turbulência, separação de fluxo, re-fixação, impacto).

Essa complexidade de dados pode ser reduzida através de modelagem orientada por dados^6 - 8^ e por algoritmos de detecção de anomalias (amplamente utilizados na análise de ECG),^9^ que utilizam redes neurais profundas para localizar e destacar valores discrepantes nos dados de fluxo específicos do paciente para orientar o clínico em relação a potenciais anomalias.

Almeida et al.,^2^ abordou os problemas visualizando estruturas coerentes lagrangeanas^10^ no campo de fluxo e computando valores escalares únicos que caracterizam aspectos específicos do campo de fluxo (por exemplo, índice de helicidade). Outros propuseram métricas para caracterizar a interação biomecânica entre o fluxo sanguíneo e as paredes arteriais^11 , 12^ ou o efeito do fluxo de cisalhamento no trauma sanguíneo e na trombogenicidade.^13 , 14^ É de suma importância estabelecer o valor clínico de tais métricas e estabelecê-las como marcadores diagnósticos ou escores clínicos. Somente com tal abordagem possibilitaremos uma interpretação eficiente e padronizada dos dados de CFD na rotina clínica.

## Infraestrutura de TI integrada

A integração total da CFD no fluxo de trabalho clínico requer interfaces de transferência de dados fáceis de usar entre bancos de dados de pacientes clínicos, sistemas de imagem e plataformas computacionais para realizar a CFD. Em casos complexos, com maior demanda por poder computacional, os dados de imagem podem necessitar ser transferidos para infraestruturas computacionais centralizadas, que podem estar fora do perímetro de TI do hospital. Isso levanta questões sobre privacidade e segurança de dados, que devem ser abordadas estabelecendo tecnologia de criptografia apropriada e conexões dedicadas. Os serviços externos também devem considerar os aspectos regulatórios da transferência de dados de pacientes pela internet. Vale a pena procurar soluções utilizadas em aplicativos computacionais estabelecidos anteriormente (por exemplo, FFR_CT_).^15^

## Engenheiro Clínico

O sucesso no enfrentamento desses problemas requer o estabelecimento do papel de um engenheiro clínico que faz parte da equipe de radiologia clínica e está totalmente integrado ao fluxo de trabalho. Só assim, a CFD tem potencial para encontrar seu lugar como um recurso diagnóstico sustentável na rotina clínica.

Com essas medidas propostas, a CFD acabará se tornando apenas mais uma modalidade dentro de um ecossistema radiológico multimodal integrado, fornecendo dados adicionais para o especialista clínico obter melhor diagnóstico e previsão, incluindo a estratificação de risco adaptada individualmente.
