# Supplementary material for: Fluidodinâmica Computacional (CFD) para Prever Alterações Patológicas na Aorta: Está Pronta para Uso Clínico?
Source: Arq Bras Cardiol. 2022 Feb 14;118(2):461–2. [Article in Portuguese] doi: 10.36660/abc.20220040 (PMC8856675; doi:10.36660/abc.20220040)
Supplement: Translation

# Translation: Computational Fluid Dynamics (CFD) For Predicting Pathological Changes In The Aorta: Is It Ready For Clinical Use?

**Authors:** Dominik Obrist, Hendrik von Tengg-Kobligk

**Affiliations:** 1 Switzerland ARTORG Center for Biomedical Engineering Research, University of Bern, Bern – Switzerland; 2 Switzerland Institute for Diagnostic, Interventional and Pediatric Radiology (DIPR), University of Bern, Bern – Switzerland

**Keywords:** Cardiovascular Diseases/phyiopathology, Cardiovascular Diseases/diagnosis, Computer Simulation, Image Processing, Computer Assisted, Prosthesis Design, Prosthesis Implantation, Biomedical Engineering

Computational modelling of complex flow problems profits from a mature basis of Computational Fluid Dynamics (CFD) software developed over the past decades. Early use of CFD was limited to academic research. Nowadays, it is a fully established tool in many industrial applications (e.g., automotive, aerospace), although CFD remains to be one of the most demanding computational tasks and the study of complex flow problems is often limited by the available computational power. The continuous exponential increase in computational power and the improved accessibility of high-performance computing infrastructure was an enabler for the ever-growing use of CFD.

Under the light of this success, it is surprising that CFD can hardly be found in clinical practice. Despite remarkable progress in modelling complex blood flow and in identifying quantitative markers for pathological flow patterns,^1^ most biomedical applications of CFD remain at the level of academic research and single-patient cases. The study by Almeida et al.^2^ is one of the few CFD studies using longitudinal radiological data from patient cohorts. The proposed prediction of pathological changes in the aorta based on CFD illustrates the potential of this technology to become an established diagnostic modality. Some of the challenges successfully addressed in this study are exemplary for the reason why CFD has not yet found its place in clinical routine. We can identify four problem fields: a) The difficulty of efficiently generating patient-specific CFD models; b) The need for reduction of data complexity to make CFD results accessible to the clinician; c) The missing IT infrastructure which integrates CFD tools smoothly into existing clinical data workflows; d) The lack of experts in the clinical environment, i.e. a clinical engineer supporting the caring physicians. In the following, we will discuss these problems and indicate possible measures to mitigate them.

## Patient-specific modelling

Patient-specific models^3^ have been successfully deployed by many researchers. Nevertheless, the translation of high-resolution radiological data into geometrical vascular models for CFD remains a time-intensive task which is often cumbersome and requires trained specialists. There is a lack of automated segmentation and meshing tools for biomedical tasks. Recent developments based on deep neural networks may provide acceptable solutions for clinical use.^4^

The setting of boundary conditions (e.g., velocity profiles at inflow/outflow) requires great care, because boundary conditions have a strong effect on the quality of the results. Therefore, it is useful to include flow measurements at well-defined locations (e.g., 2D+time or 3D+time blood flow MRI) into radiologic protocols.^5^ Next to the adequate choice of the modality and body location, this requires appropriate temporal and spatial resolution of the scans.

Furthermore, we need a better understanding of diseased tissue biomechanics to correctly interpret a remodeled or dissected aorta or the plaque on a vessel wall. Next to continued classical biomechanical research, this requires studies with large cohorts including healthy volunteers to understand the clinical relevance of biomechanical models.

## Diagnostic markers

Analysis of CFD results is challenging even for experts in fluid dynamics. This is partly due to the difficulty of visualizing three-dimensional, time-dependent flow fields featuring a wealth of flow phenomena which may be relevant for the clinical interpretation of the results (e.g., vortices, flow instabilities, turbulence, flow separation, re-attachment, impingement).

This data complexity can be reduced by data-driven modelling^6 - 8^ and by anomaly detection algorithms (used widely in ECG analysis^9^ ) which use deep neural networks to localize and highlight outliers in the patient-specific flow data to guide the clinician toward potential anomalies.

Almeida et al.^2^ addressed the problems by visualizing Lagrangian coherent structures^10^ in the flow field and by computing single scalar values that characterize specific aspects of the flow field (e.g. helicity index). Others proposed metrics to characterize the biomechanical interaction between blood flow and arterial walls^11 , 12^ or the effect of shear flow on blood trauma and thrombogenicity.^13 , 14^ It is of paramount importance to establish the clinical value of such metrics and to establish them as diagnostic markers or clinical scores. Only with such an approach we will enable efficient and standardized interpretation of CFD data in clinical routine.

## Integrated IT infrastructure

Full integration of CFD into the clinical workflow requires easy-to-use data transfer interfaces between clinical patient databases, imaging systems and computational platforms to perform CFD. In complex cases with higher demand for computational power, imaging data may have to be transferred to centralized computational infrastructure which may be outside of the hospital’s IT perimeter. This raises questions of data privacy and security which must be addressed by establishing appropriate encryption technology and dedicated connections. External services must also consider regulatory aspects of transferring patient data over the internet. It will be worthwhile to look at solutions used in already established computational applications (e.g. FFR_CT_^15^ ).

## Clinical Engineer

Successful tackling of these problems requires establishing the role of a clinical engineer who is part of the clinical radiology team and fully integrated in the workflow. Only then, CFD has the potential to find its place as a sustainable diagnostic resource in clinical routine.

With these proposed measures, CFD will eventually become just another modality within an integrated multi-modal radiologic ecosystem providing additional cues to the clinical expert to arrive at a better diagnosis and prediction including individually adapted risk stratification.
